# A mutagenesis-derived broad-spectrum disease resistance locus in wheat

**DOI:** 10.1007/s00122-012-1841-7

**Published:** 2012-03-25

**Authors:** Jackie Campbell, Hongtao Zhang, Michael J. Giroux, Leila Feiz, Yue Jin, Meinan Wang, Xianming Chen, Li Huang

**Affiliations:** 1 Department of Plant Sciences and Plant Pathology, Montana State University, Bozeman, MT 59717-3150 USA; 2 Cereal Disease Laboratory, United States Department of Agriculture-Agricultural Research Service (USDA-ARS), St. Paul, MN 55108 USA; 3 Wheat Genetics, Physiology, Quality, and Disease Research Unit, United States Department of Agriculture-Agricultural Research Service (USDA-ARS), Pullman, WA 99164-6430 USA; 4 Present Address: The Boyce Thompson Institute for Plant Research, Ithaca, NY 14853-1801 USA; 5 Department of Plant Pathology, Washington State University, Pullman, WA 99164-6430 USA

## Abstract

Wheat leaf rust, stem rust, stripe rust, and powdery mildew caused by the fungal pathogens *Puccinia triticina*, *P. graminis* f. sp. *tritici*, *P. striiformis* f. sp. *tritici*, and *Blumeria graminis* f. sp. *tritici*, respectively, are destructive diseases of wheat worldwide. Breeding durable disease resistance cultivars rely largely on continually introgressing new resistance genes, especially the genes with different defense mechanisms, into adapted varieties. Here, we describe a new resistance gene obtained by mutagenesis. The mutant, MNR220 (*m*utagenesis-derived *n*ew *r*esistance), enhances resistance to three rusts and powdery mildew, with the characteristics of delayed disease development at the seedling stage and completed resistance at the adult plant stage. Genetic analysis demonstrated that the resistance in MNR220 is conferred by a single semidominant gene mapped on the short arm of chromosome 2B. Gene expression profiling of several pathogenesis-related genes indicated that MNR220 has an elevated and rapid pathogen-induced response. In addition to its potential use in breeding for resistance to multiple diseases, high-resolution mapping and cloning of the disease resistance locus in MNR220 may lead to a better understanding of the regulation of defense responses in wheat.

## Introduction

Bread wheat (*Triticum aestivum* L.) provides approximately 40 % of the food and 25 % of the calories consumed globally (Board 2010). Bread wheat is an allohexaploid (2*n* = 6*x* = 42) consisting of three related genomes designated A, B, and D with an estimated genome size of 16,000 Mb (Arumuganathan and Earle 1991). This complex species arose from two successive hybridization and speciation events involving domesticated and wild grasses (Dvořák et al. 1993; McFadden and Sears 1964; Sarkar and Stebbins 1956). The relatively recent speciation and domestication history of wheat led to limited genetic variation within the species.

Modern wheat improvement efforts are centered on yield and increased resistance to biotic stresses, especially fungal pathogens. Disease resistance can be accomplished either through passive or active host defense mechanisms. Passive defense involves physical or chemical barriers to infection such as the cuticle and phytoanticipins presented by the host without direct pathogen recognition. Phytoanticipins are constitutively expressed tissue-specific antimicrobial chemicals (Bouarab et al. 2002) such as defensins that are found in the wheat endosperms (Colilla et al. 1990). Active defense, such as the hypersensitive response (HR), involves physiological changes in the host induced by pathogen recognition. Active defense involves signaling pathways such as those mediated by jasmonic acid (JA) and salicylic acid (SA). The SA signaling pathway is mainly induced by biotrophic pathogens and is correlated with high expression of specific *PR* genes (Glazebrook 2005).

The three wheat rusts are leaf rust caused by *Puccinia triticina* (*Pt*), stem rust caused by *P. graminis* f. sp. *tritici* (*Pgt*), and stripe rust caused by *P. striiformis* f. sp. *tritici* (*Pst*). Powdery mildew is caused by *Blumeria graminis* f. sp. *tritici* (*Bgt*). Rusts and powdery mildew are biotrophs capable of causing large scale epidemics. Breeding resistance to these diseases is a major undertaking in wheat improvement worldwide. Types of rust and powdery mildew resistances are characterized as race specific or non-specific based on the mode of action. Race-specific resistance involves a gene-for-gene interaction between the host and pathogen (Flor 1956). This interaction depends on host recognition of pathogen effectors, or avirulence factors. This type of resistance is often controlled by a single host resistance gene (*R*-gene) that triggers programed cell death at the infection sites. Although race-specific resistance is often highly effective, it tends to be short-lived as host recognition is easily overcome by a change in or the deletion of the corresponding pathogen effector. Alternatively, race non-specific resistance is primarily conferred by multiple genes with intermediate or additive effects. As race non-specific resistance is not dependent upon a single gene-specific interaction it tends to be more durable. This type of resistance does not rely upon HR and confers resistance through retardation of disease development by a longer latent period, lower infection frequency, and/or reduced sporulation (Rubiales and Niks 1995).

*R*-gene mediated resistance has been used by wheat improvement programs for decades to control wheat rust diseases. However, only relatively small set of effective, rust *R* genes deployed among cultivated varieties place a high selective pressure on pathogen populations. The selective pressure can lead to emergence of pathogen variants that avoid recognition by deployed *R* genes, and hence are capable of causing devastating disease epidemics. One of the most recent incidents occurred in 1999 when *Pgt* race TTKSK (or Ug99) was first identified in Uganda and subsequently caused stem rust epidemics in Kenya (Pretorius et al. 2000; Stokstad 2007; Wanyera et al. 2006). Hence, breeding for rust resistance involves the continual introgression of new resistance genes into adapted cultivars. Here, we describe a wheat mutant identified from an ethylmethane sulfonate (EMS) mutagenized population created in the spring wheat cultivar Alpowa (PI 566596) that exhibits dominantly inherited resistance to the three rusts and powdery mildew.

## Materials and methods

### Materials

**Fig. 1:**
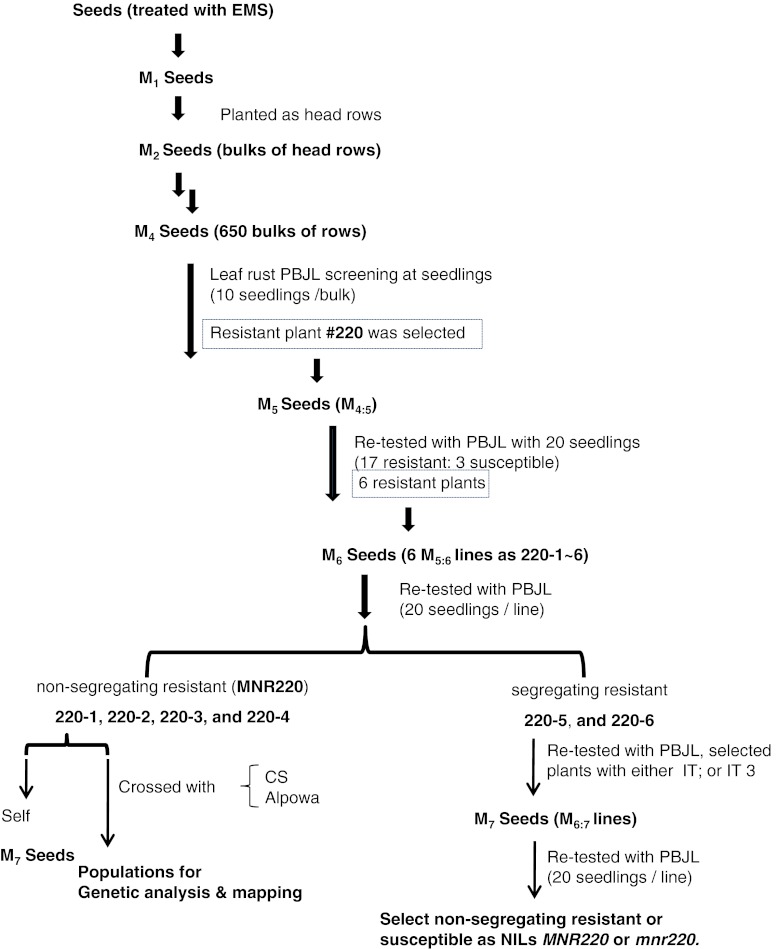
A scheme showing steps of mutant identification, confirmation and genetic analysis. M_*n*:*n* + 1_ line is used to describe *n*_+1_ generation seeds that derived from one selected *n* generation plant

Plant materials Alpowa (PI 566596) and McNair 701 (CItr 15288) were obtained from the USDA National Plant Germplasm System (NPGS). Chinese Spring was provided by Dr. Luther Talbert at Montana State University. Avocet Susceptible (AvS) is an Australia spring wheat cultivar originally provided by Dr. Colin Wellings, Plant Breeding Institute, University of Sydney. Identification of MNR220 and development of the near isogenic lines (NILs) were outlined in Fig. 1. Resistant and susceptible NILs were developed after eight generations of selfing from one *M*_7_ heterozygous line 220-5. MNR220 heterozygous lines were selected at each generation with IT 2 to PBJL and confirmed with progeny tests of 20 seedlings.

Leaf rust isolate PBJL was provided by Dr. Robert Bowden from USDA-ARS, Manhattan, KS. The other two leaf rust isolates SBDGD and BBBDD and one stripe rust isolate PST-139 were collected in Creston, MT. Stem rust isolates were from the collection of the Cereal Disease Laboratory (CDL), St. Paul, MN. Stripe rust isolates were from the USDA-ARS, Wheat Genetics, Quality, Physiology and Disease Research Unit, Pullman, WA.

## Methods

### EMS-induced mutagenesis

The mutagenized population screened here was created by EMS seed treatment of the soft, white, spring wheat cultivar Alpowa (Feiz et al. 2009).

#### Plant growth

Seeds were germinated in 164 mL cone-tainers (Stuewe & Sons Inc., Tangent, OR) filled with 50:50 Mix with half of MSU Mix and half Sunshine Mix #1 by volume. Seedlings were grown in the MSU Plant Growth Center (MSU-PGC) greenhouse with 22/14 °C day/night temperatures and a 16-h photoperiod. Plants were watered as needed and fertilized every other day with Peters General Purpose Plant Food (Scotts-Miracle-Gro Company, Marysville, OH).

### Rust inoculation and disease assessment

#### Leaf rust inoculation and disease assessment

Leaf rust tests were conducted at MSU-PGC. Controlled greenhouse inoculations were performed on seedlings and adult plants. Each inoculation was conducted using race PBJL of *P. triticina*. The inoculum consisted of urediniospores suspended in Soltrol 170 Isoparaffin (Chempoint, Bellevue, WA). The spore inoculum density was calculated at 227,500 spores/ml using a Brightline hemocytometer as per the manufacturer’s recommendations (Hausser Scientific, Horsham, PA). The inoculum was applied at a rate of 0.05 mg spores/10 μl Soltrol/plant using a Badger 350-3 airbrush gun (Badger Air-Brush Co., Franklin Park, IL). Spore germination rate was assessed on an inoculated microscope slide using a light microscope. Inoculated plants were immediately transferred to a Percival I-60D dew chamber (Percival Scientific Inc., Perry, IA) pre-conditioned to an air temperature of 15–17 °C and incubated for 24 h. The plants were then transferred to the greenhouse bench and grown under the conditions described above. Disease responses were assessed when rust symptoms were fully expressed on Alpowa 8–22 dpi using the seedling 0–4 IT scale (McIntosh et al. 1995; Stakman et al. 1962). In details, IT 0: no visible uredia; IT;: hypersensitive flecks; IT 1: small uredia with necrosis; IT 2: small- to medium-sized uredia with green islands surrounded by necrosis; IT 3: medium-sized uredia without necrosis; IT 4: large-sized uredia without necrosis. The variations within each class are indicated by the use of − (less than average for the class) and + (more than average for the class). When variable reactions were observed, IT ranges are listed from lowest to highest.

#### Stripe rust inoculations and disease assessments

The stripe rust nursery at the Northwestern Agricultural Research Station, Creston, MT was used for adult plant stripe rust assessments. The materials were planted in triplicated three hill plots 2 m apart. Stripe rust was assessed at anthesis stage based on the disease severity scales described by McIntosh et al. (1995).

Additional stripe rust assessments were conducted under controlled greenhouse conditions in Pullman, WA. Races PST-78 and PST-127 were used separately in seedling and adult plant tests. About ten plants were used in each test. For the seedling tests, plants of Alpowa, MNR220 and AvS (used as a susceptible check) at the two-leaf stage were dust inoculated with a mixture of urediniospores and talc (Fisher, Pittsburgh, PA) in a ratio of 1:20 and incubated in a dew chamber at 10 °C without light for 24 h. The inoculated seedlings were grown in a growth chamber with a diurnal temperature cycle gradually changing from 4 °C at 2:00 am to 20 °C at 2:00 pm with a 16-h photoperiod in each cycle. For the adult plant tests, plants at booting were inoculated, incubated in a dew chamber, and grown in a growth chamber in the same way as for the seedling tests, except with a diurnal temperature cycle gradually changing from 10 °C at 2:00 am to 30 °C at 2:00 pm. Stripe rust infection types were assessed based on a 0 (immune)–9 scale (highest susceptible) (Line and Qayoum 1992).

#### Stem rust inoculation and disease assessment

Stem rust assessments were conducted at both the MSU-PGC and the University of Minnesota. Tests with QFCSC and TLMKC were conducted at MSU-PGC. Inoculations were conducted in a similar manner to leaf rust with the following exceptions: the dew chamber was pre-conditioned to an air temperature of 19–22 °C and incubated for 24 h, followed by incubation under high humidity and light intensity conditions for at least 3 h before being transferred to the greenhouse. Assessments were made when Alpowa showed full susceptibility 10–28 dpi using a modified Stakman et al. (1962) 0–4 IT scale (McIntosh et al. 1995).

Stem rust tests with additional races including TTKSK (Ug99) and its variants were conducted at the USDA/University of Minnesota St Paul, MN, Disease Laboratory. Inoculation procedures and disease assessment were as described (Jin et al. 2007).

### Genetic analysis

For genetic analysis, homozygous mutant MNR220 (220-1) (Fig. 1) at M_6_ was crossed with Alpowa (as male parent) and Chinese Spring (as female parent). Ten F_1_ individuals each from the two crosses were tested as resistant to PBJL and selfed to produce F_2_ seeds with the possibility of outcrossing being prevented by the bagging of heads prior to anthesis. The 281 F_2_ individuals used for segregation analysis were from a combination of three F_1:2_ populations between MNR220 and Alpowa, and all 136 F_2_ individuals between Chinese Spring and MNR220 were from one F_1:2_ population. F_2_ seedlings were inoculated with PBJL and ITs were assessed on a single plant at 8, 10 and 12 dpi three time points during disease development.

In order to assess whether the same locus confers the resistance to leaf rust, stem rust and powdery mildew, we tested the three pathogens on each of the eight F_2:3_ lines from MNR220/Alpowa and 98 F_2:3_ lines from Chinese spring/MNR220 with three different sets of 20 seedlings each from the same F_2:3_ line. If all three sets of 20 seedlings were not segregating or all three were segregating to the *Pt*, *Pst* and *Bgt* races, we describe the resistance to the three pathogens was co-segregating in this F_2:3_ line.

### Genomic DNA isolation and marker analysis

A cross between Chinese Spring and MNR220 was used for genetic mapping of the *MNR220* locus. Leaf tissues for genomic DNAs were collected from the parents and F_2_ individual plants but genotypes of the F_2_ plants were based on the tests of 20 seedlings from each of the F_2:3_ lines. Genomic DNAs were isolated using the QIAGEN DNeasy Plant Mini Kit (Qiagen Sciences Inc, Germantown, MD). For bulked segregant analysis (Michelmore et al. 1991), two resistant and two susceptible DNA bulks were assembled using equal amounts of DNA from 5 or 10 homozygous resistant and 5 or 10 homozygous susceptible F_2_ plants, respectively. Wheat simple sequence repeat (SSR) markers (*Xgwm*, *Xwmc*, *Xbarc*, *Xcfa*, *Xcfd* and *Xcfp* series) from 21 wheat chromosomes and some of the wheat EST-STS markers located on chromosome 2BS were used for screening polymorphisms between the two parents. Sequences of the primers of these SSR and EST-STS markers are available from GrainGenes2.0 website http://wheat.pw.usda.gov and http://wheat.pw.usda.gov/SNP/primers/contig_primer_list.xls. Then, the polymorphic markers between the parents were used to screen the resistant and susceptible bulks. The markers revealed the same polymorphic patterns between the parents and the resistant and susceptible bulks were tested in the entire population.

PCR amplifications were conducted in 20 μl reactions containing 20 mM Tris–HCl, pH 8.3, 100 mM KCl, 3.0 mM MgCl_2_, 0.4 mM dNTP, 50 ng of each primer, 100 ng genomic DNA and 1.5 U Taq DNA polymerase. Amplifications were performed at 94 °C for 5 min, followed by 40 cycles at 94 °C for 45 s, 50–60 °C (depending on specific primers) for 45 s, and 72 °C for 30 s–1 min (depending on different primers), with a final extension at 72 °C for 10 min. PCR products were mixed with 3 μl loading buffer (98 % formamide, 10 mM EDTA, 0.25 % bromophenol blue, and 0.25 % xylene cyanol) and separated in 8 or 12 % non-denaturing polyacrylamide gels (39:1 acrylamide:bisacrylamide) (EMD Chemicals Inc, Gibbstown, NJ) and stained by GelRed (Bio-Rad, Hercules, CA).

### Data analysis and genetic mapping

Chi-squared test (χ^2^) was used to evaluate deviations of observed data from theoretically expected segregation ratios. Linkages between molecular markers and the resistance gene were determined using Mapmaker 3.0b (Lincoln et al. 1992) with an LOD score of 3.0 as the threshold. The genetic map was drawn with the software Mapdraw V2.1 (Liu and Meng 2003).

### Transcript abundance analysis by RT-qPCR

In order to assess the transcript abundances of seven *PR* genes, relative quantitative reverse transcriptase polymerase chain reaction (RT-qPCR) and gene-specific primers from Desmond et al. (2008), designed based on wheat sequences, were used. Inoculation with Soltrol 170 was used as mock control. Leaf tissues were collected at 0, 0.5, 1, 1.5 and 5.5 dpi with PBJL and stored at −80 °C until RNA isolation. Total RNA was isolated and treated with DNase I on column using the Qiagen RNeasy Plant Mini Kit (Qiagen, Valencia, CA) as per the manufacturer’s suggestions. The quality and concentration of total RNA were assessed via agarose gels and 260/280_ABS_ measurements on a NanoDrop 1000 spectrophotometer (Thermo Fisher Scientific Inc., Wilmington, DE). To exclude contamination with genomic DNA (gDNA) each RNA sample was used as template in a reverse transcriptase-free PCR reaction using the Actin (ACT) control primers specific to wheat as per the manufacturer’s protocol. The same volume and concentration of the normalized RNA was used as template in each reaction as normalized ACT transcript abundance using iScript One-Step RT-PCR Kit with SYBR Green (Bio-Rad, Herculeqs, CA) as per the manufacturer’s recommended protocol. Transcript abundance was quantified via real time PCR on a CFX96 real time PCR detection system (Bio-Rad, Hercules, CA) using the iScript One-Step RT-PCR Kit with SYBR Green (Bio-Rad, Hercules, CA) and gene-specific primers. Each reaction was conducted in triplicate of the two biological replicates and data were used only if the standard deviation between replicates was ≤0.3 *C*_t_. The ACT gene-specific primers are ACT forward.

5′AAATCTGGCATCACACTTTCTAC3′ and ACT reverse 5′GTCTCAAACATATCTGGGTCATC3′, amplified a 127-bp product of the coding sequence of the gene. All *PR* gene primer sets were based on Desmond et al. 2008. Transcript abundance was calculated with the threshold cycle (*C*_t_) using the ΔΔ*C*_t_ method as described in the CFX96 manual (Bio-Rad, Hercules, CA), where fold change = 2 − ΔΔ*C*_t_ and percent transcript abundance = fold change × 100. The transcript abundances for each group were calculated as the average plus or minus the average difference between the average and the highest and lowest values in that group. All statistical analyses were conducted using Microsoft Excel (Microsoft Corp., Redmond, WA).

## Results

### Creation and identification of a new resistance gene

The EMS-mutagenized population used in this study (Feiz et al. 2009) was created using the soft white spring wheat cultivar Alpowa. Initial rust screening was done at the M_4_ generation (Fig. 1). In detail, approximately 10 seedlings from each of 650 M_4_ families were inoculated with *Pt* race PBJL. Response to the pathogen was assessed 8 days post-inoculation (dpi) and described as infection type (IT) on a 0 (immune) to 4 (susceptible) scale (McIntosh et al. 1995). From the ~6,500 individual plants tested, 11 individual plants from different M_4_ families were selected based on their enhanced resistance compared to wild-type Alpowa. The selected plants showed moderate resistance characterized by small pustules surrounded by necrosis with IT 22^+^ and were rated as resistant. Alpowa had medium-sized pustules without necrosis and was rated as susceptible with IT 3. The resistance of each of the four selected plants was confirmed by M_4:5_ progeny tests using the same *Pt* race on 20 M_5_ seedlings. Here, we focus on one mutant line selected from 10 seedlings of a single M_4_ family labeled as #220 in the mutagenized Alpowa population of Feiz et al. (2009). The single plant was selfed at the M_4_ generation and the 20 M_4:5_ seedlings were tested with PBJL, showing a segregation ratio of 17 resistant to 3 susceptible. Among the 20 seedlings, six resistant individuals labeled as 220-1 to 6 with IT 1-2 were selfed. Twenty seedlings from each of the M_5:6_ were tested with PBJL, four M_5:6_ lines (220-1, 220-2, 220-3, 220-4) showed non-segregating resistance with IT;1 on all 20 seedlings, indicating they were homozygous for PBJL resistance (Fig. 1). Hereafter, we designate the homozygous resistant plants derived from the #220-1 as mutant MNR220 (*M*utagenesis-derived *N*ew *R*esistance) and the mutated allele as *MNR220*.

In addition to leaf rust tests, different seedlings of 220-1 M_5:6_ line were tested with 13 *Pgt* races representing diverse virulence combinations, including races in the Ug99 lineage (Jin et al. 2008, 2009). At the seedling stage, Alpowa and MNR220 showed similar ITs to RCRSC (IT 2^−^), RKQQC (IT;1^+^), and QCCSM (IT 22^+^) (Table 1), suggesting that Alpowa was resistant to these three races. MNR220 showed moderately lower ITs to TRTTF (IT 12) and MCCFC (IT;1) compared to Alpowa, suggesting the mutant has enhanced resistance to these two races (Table 1). Alpowa exhibited ITs 34 to TLMKC, QFCSC, TPMKC, TTKSK, TTKST, and TTTSK, whereas MNR220 had distinctly low ITs;1^+^ to these races.

**Table 1:** Infection types (IT) of MNR220, Alpowa and McNair 701 challenged with races of *Puccinia tritici* (*Pt*), *P. striiformis* f. sp. *tritici* (*Pst*), and *P. graminis* f. sp. *tritici* (*Pgt*)

Pathogen	Race^b^	Growth stage	Infection type^a^		
			Alpowa	MNR220	McNair 701
*Pt*	PBJL	Seedling^c^	3^+^4	;2	3^+^
		Adult^c^	S	R	S
	SBDGD	Adult^d^	S	I	
	BBBDD	Adult^d^	S	I	
*Pst*	PST-78	Seedling^e^	8	7	
	PST-127	Seedling^e^	8	8	
	PST-127	Adult^e^	2	2	
	PST-127, PST-139	Adult^d^	S	R	
*Pgt*	TLMKC	Seedling^c^	3^−^4	;1^+^	3^+^4
	QFCSC	Seedling^c^	34	;1^+^	3^+^4
	TTKSK	Seedling^f^	3^-^3^+^	;	4
	TTKST	Seedling^f^	3^+^	;1^+^	4
	TTTSK	Seedling^f^	3^+^	;	4
	TRTTF	Seedling^f^	22^+^	12	2^+^3^−^
	QTHJC	Seedling^f^	3^+^	3	4
	MCCFC	Seedling^f^	;2	;1	4
	RCRSC	Seedling^f^	2^−^	2^−^	4
	RKQQC	Seedling^f^	;1^+^	;	4
	TPMKC	Seedling^f^	4	;	4
	TTTTF	Seedling^f^	4	3^+^	4
	QCCSM	Seedling^f^	2^+^	2	4

^a^Infection types indicated as per 0 (immune)–4 (susceptible) scale was used for *Pt* and per 0 (immune)–9 (susceptible) scale for *Pst* as described in McIntosh et al. (1995). *0* no visible uredia,; hypersensitive flecks, *1* small uredia with necrosis, *2* small- to medium-sized uredia with green islands and surrounded by necrosis, *3* medium-sized uredia without necrosis, *4* large-sized uredia without necrosis. The variations in each class are indicated by the use of − (less than average for the class) and + (more than average for the class). When variable reactions were observed IT ranges are listed from lowest to highest

^b^ *Pt* race designation as per accepted nomenclature. The *Pst* races designations as per accepted nomenclature. *Pgt* race designation as per accepted nomenclature

^c^Assessment of disease produced from controlled pathogen inoculations conducted in the MSU-PGC

^d^Assessment of disease produced from exposure to native pathogen populations in the field at Creston, MT

^e^Assessment of disease produced from controlled inoculations conducted in the greenhouse at Pullman, WA

^f^Assessment of disease produced from controlled pathogen inoculations conducted in the contained laboratory at St Paul, MN; *Pgt* race Ug99 is the newly identified race and current focus of stem rust resistance breeding, therefore, the five-letter race identifications of Ug99 (TTKSK) and its derivatives are highlighted in gray

MNR220 was also tested with two *Pst* races at the seedling and one *Pst* race at the adult plant stages. At the seedling stage, MNR220 and Alpowa showed similar susceptible ITs to *Pst* races PST-78 (IT 7 and 8) and PST-127 (IT 8) (Table 1). Additionally, at the adult plant stage, MNR220 and Alpowa showed similar resistant ITs 2 to PST-127 (Table 1).

The M_6_ generation of MNR220 and Alpowa was planted at one field location in Creston, Montana in 2010 with three replications (3 hill plots/replication) for disease assessments at the adult plant stage to local *Pt* and *Pst* populations. The predominant *Pt* races were SBDGD and BBBDD and the *Pst* races were PST-127 and PST-139 (Wang and Chen, unpublished data). MNR220 showed an immune response to leaf rust and a resistance response to stripe rust, whereas Alpowa was susceptible to both diseases in the field (Table 1).

### Characteristics of the defense responses of MNR220 at the seedling and the adult plant stages

**Fig. 2:**
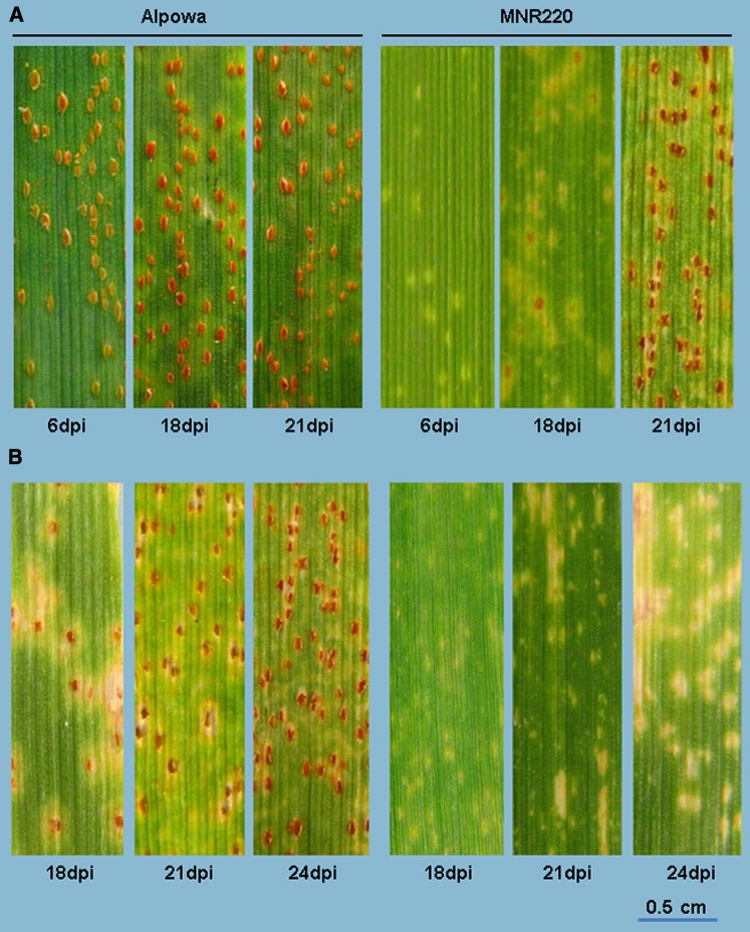
Leaf rust disease development at three time points post-inoculation with *P. triticina* race PBJL. **a** Infection types at the seedling stage. **b** Infection types at the adult plant stage

The response of MNR220 to *Pt* race PBJL was dosage dependent and developmentally specific. At the seedling stage, both Alpowa and MNR220 showed necrosis at infection sites 3–4 dpi, but by 6 dpi the rust on Alpowa was sporulating, whereas MNR220 had only necrosis flecks (Fig. 2a). The seedling low infection type normally can last until 18 dpi (Fig. 2a). The delayed sporulation observed on MNR220 at seedling stage became insignificant after 18 dpi when the infection types on MNR220 were the same as on Alpowa (Fig. 2a). However, when tested at the adult plant stage, infection and disease development on Alpowa mirrored seedling stage inoculations, whereas MNR220 showed only HR-like flecks from 6 dpi until leaf senescence (around 35 dpi, data not shown). Figure 2b only shows the infection types up to 24 dpi.

**Fig. 3:**
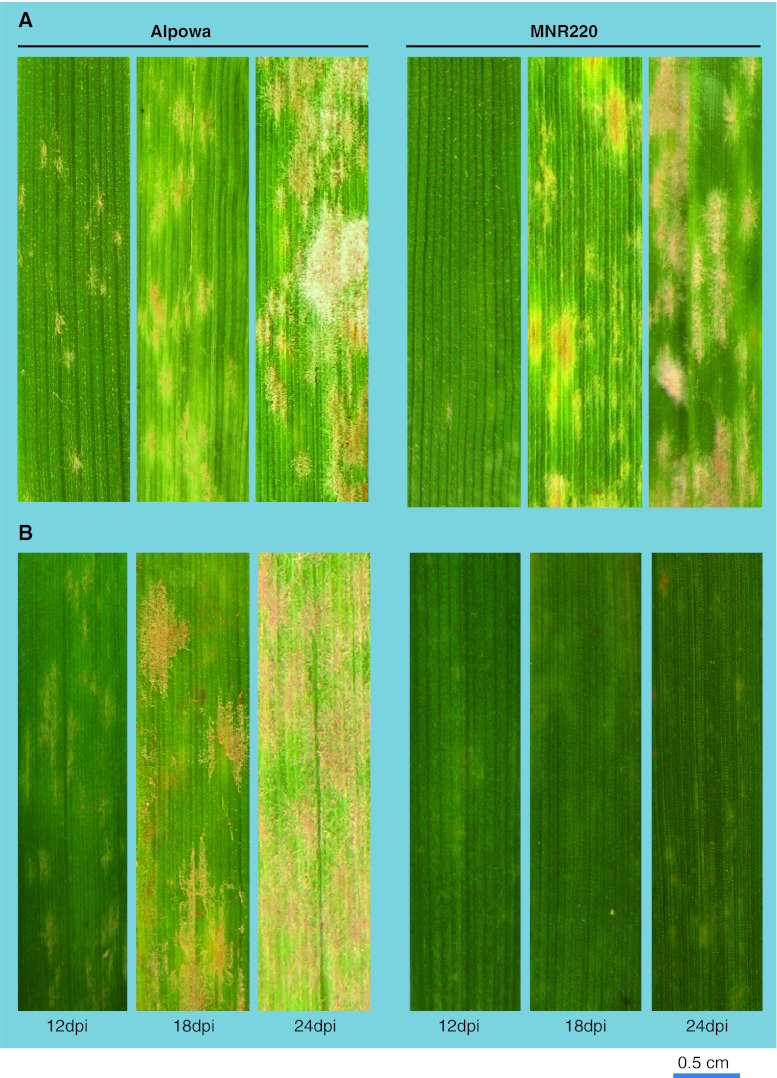
Powdery mildew disease development at three time points post-inoculation with *B. graminis*. **a** Infection types at the seedling stage. **b.** Infection types at the adult plant stage

In order to evaluate the response spectra to another fungal pathogen, MNR220 was inoculated with a mixture of *Bgt* (unknown race identification) collected from the Plant Growth Center at Montana State University. The mutant’s response to powdery mildew was similar to that observed in response to leaf rust, with the characteristics of delayed disease symptoms at the seedling stage. The first post-inoculation time point when the wild type and MNR220 had visually observable difference to *Bgt* was 12 dpi when Alpowa showed patches of mycelium and conidia on the leaf surface and MNR220 had almost none (Fig. 3a); and the difference between wild type and the mutant was insignificant at 18 dpi. When inoculated with *Bgt* post-tiller development, MNR220 remained highly resistant until leaf senescence around 35 dpi, whereas Alpowa was fully susceptible (Fig. 3b).

### Morphology of MNR220

**Fig. 4:**
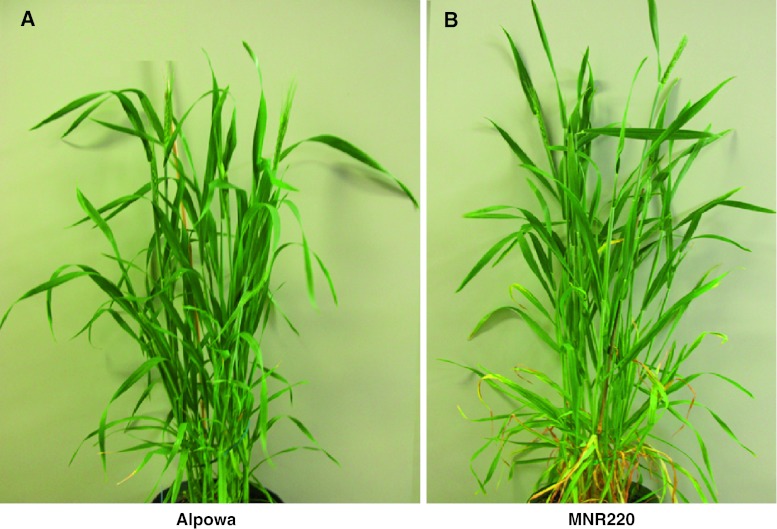
Adult plant phenotype. **a**. Alpowa and **b.** MNR220

After crossing 220-1 with Alpowa and followed by four generations of backcrossing with Alpowa and selfing, lines that carried the resistance conferred by *MNR220* had few phenotypic differences from wild-type Alpowa. Under greenhouse conditions, the advanced MNR220 lines had similar tiller numbers, maturity date, and seed morphologies as Alpowa, but leaf senescence starting from the lower leaves 5~6 days before heading was earlier than Alpowa (Fig. 4). Additionally, traits such as plant height, spike morphology, and tip sterility segregated in backcrossed and selfed MNR220 progenies. Importantly, none of the morphological differences between the mutant and the wild-type co-segregated with resistance to PBJL.

### Genetic analysis and mapping of disease resistance locus in MNR220

Crosses of M_6_ MNR220 with spring wheat cultivars Alpowa (as male parent) and Chinese Spring (CItr 14108) (as female parent) were used for genetic analysis. Ten F_1_ individuals each from the two crosses were tested as resistant to *Pt*-PBJL, indicating dominance of the mutant allele. Interestingly, F_1_ individuals from the cross of Chinese Spring/MNR220 were more resistant (IT 1) to PBJL than those of cross MNR220/Alpowa (IT 2^−^2). In addition, there were observable differences in the speed of leaf rust disease development between lines heterozygous and homozygous for the *MNR220* allele. On *MNR220* homozygous lines, small- to medium-sized uredinia developed at the infection sites by 16 dpi whereas on *MNR220* heterozygous lines, uredinia developed as early as 8 dpi. The segregation ratios of resistance to PBJL in the F_2_ populations derived from the two crosses were consistent with expected segregation at a single dominant locus (Table 2). In addition, eight selected homozygous F_2:3_ lines of MNR220/Alpowa (four resistant and four susceptible lines to *Pt* race PBJL) and 98 F_2:3_ lines of Chinese Spring/MNR220 were also tested with *Pt* race PBJL, *Pgt* race TLMKC and a mixture of unknown races of *Bgt* (20 seedlings/line/race). Resistance to *Pt, Pgt,* and *Bgt* was perfectly co-segregated among the 8 + 98 lines.

**Table 2:** Segregation analysis of seedling leaf rust resistance in the MNR220/Alpowa BC_1_F_2_ and Chinese Spring/MNR220 F_2_ populations

Cross	F_2_ individuals^a^		Expected ratio^b^	χ^2c^	*P* ^d^
	Resistant	Susceptible			
MNR220/Alpowa	210	71	3:1	0.01	0.9
Chinese Spring/MNR220	100	36	3:1	0.16	0.69

^a^Number of individuals in F_2_ populations assessed as resistant or susceptible to *Pt* race. PBJL based on 0 (immune)–4 (susceptible) infection type scale

^b^Expected Mendelian single gene segregation ratio (R:S)

^c^Calculated Chi-square (χ^2^)

^d^The likelihood that the observed segregation ratio does not fit a 3:1 ratio. There was no segregation for leaf rust resistance observed in >100 seedlings of each selfed Alpowa, or Chinese Spring

**Fig. 5:**
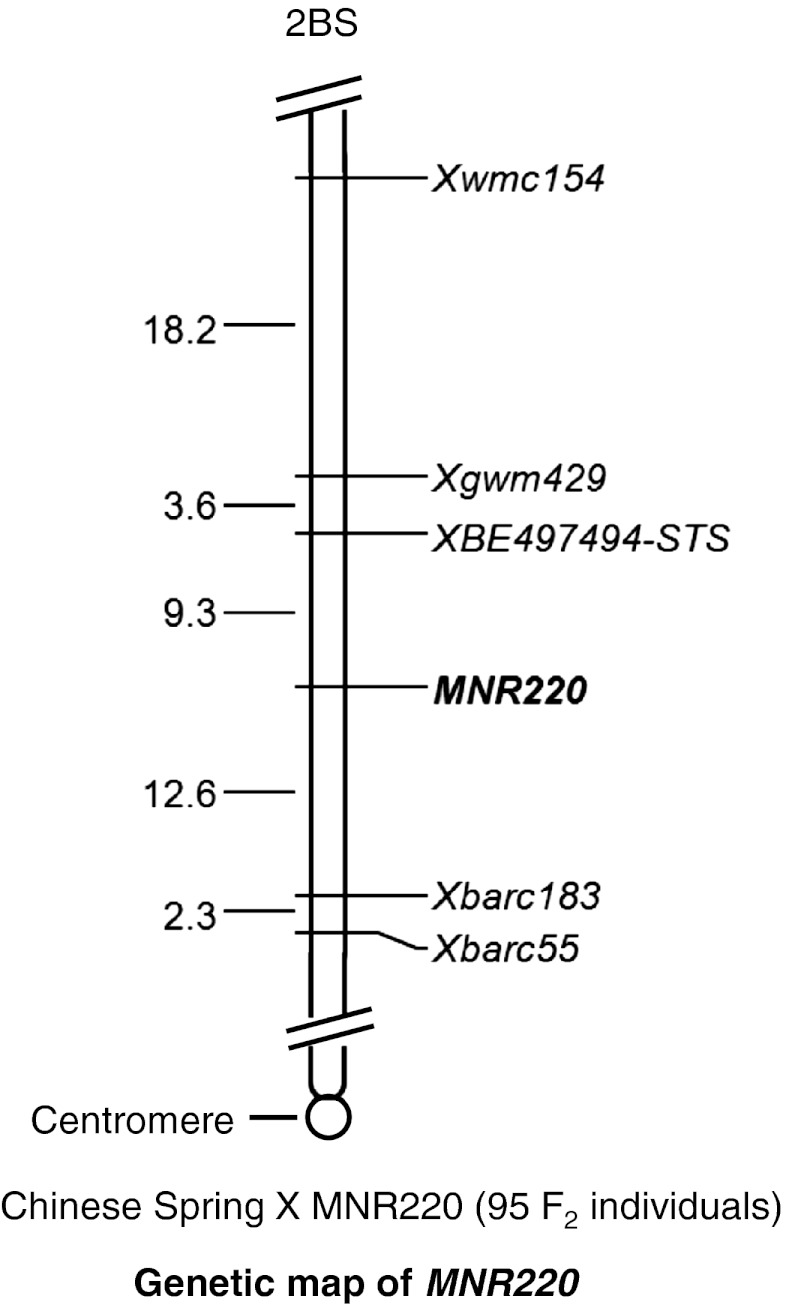
Genetic map of the *MNR220* locus constructed with the DNAs from 95 individual F_2_ plants and the genotypes from the corresponding F_2:3_ lines from a cross between Chinese Spring and MNR220

Genetic mapping of the *MNR220* locus was based on the genomic DNAs from 95 individual F_2_ plants and the genotypes of the corresponding F_2:3_ lines from a cross between Chinese Spring/MNR220. Randomly selected 358 SSR markers that cover 21 chromosomes of hexaploid wheat were used to initially screen polymorphisms between Chinese spring and MNR220. Among them, 109 markers were polymorphic between the two parents. Only two markers (*Xbarc55* and *Xwmc154*) were polymorphic between the parents and the resistant and susceptible bulks. These markers are located on the short arm of chromosome 2B. Since then, 62 more SSR markers located on 2BS were screened, two more (*Xgwm429* and *Xbarc183*) revealed the same polymorphic patterns between the parents and the bulks. These four polymorphic markers were tested in the entire population. The *MNR220* locus was placed between *Xgwm429* and *Xbarc183*. Based on the physical location of these two SSR markers, the *MNR220* locus was narrowed down to a single deletion bin 2BS-0.53–0.75 on chromosome 2B (Somyong et al. 2011). Therefore, 12 wheat EST-STS markers from that interval were used to screen polymorphism between the parents and the bulks. *XBE497494*-*STS* (developed from wheat EST BE497494) was the only marker that revealed polymorphism between the two parents and the bulks. Among the 5 polymorphic markers, 3 (*Xbarc55*, *Xwmc154*, and *Xgwm429*) were co-dominant, and 2 (*Xbarc183* and *XBE497494*-*STS*) were dominant. A linkage map including all five polymorphic markers and the *MNR220* locus was constructed and shown in Fig. 5. The *MNR220* locus is flanked by the SSR marker *Xbarc183* and the STS marker *XBE497494*-*STS*. Marker *Xbarc183* is 12.6 cM proximal to the *MNR220* locus, and *XBE497494*-*STS* is 9.3 cM distal to the locus.

### Expression profiling of pathogenesis-related (*PR*) genes

**Fig. 6:**
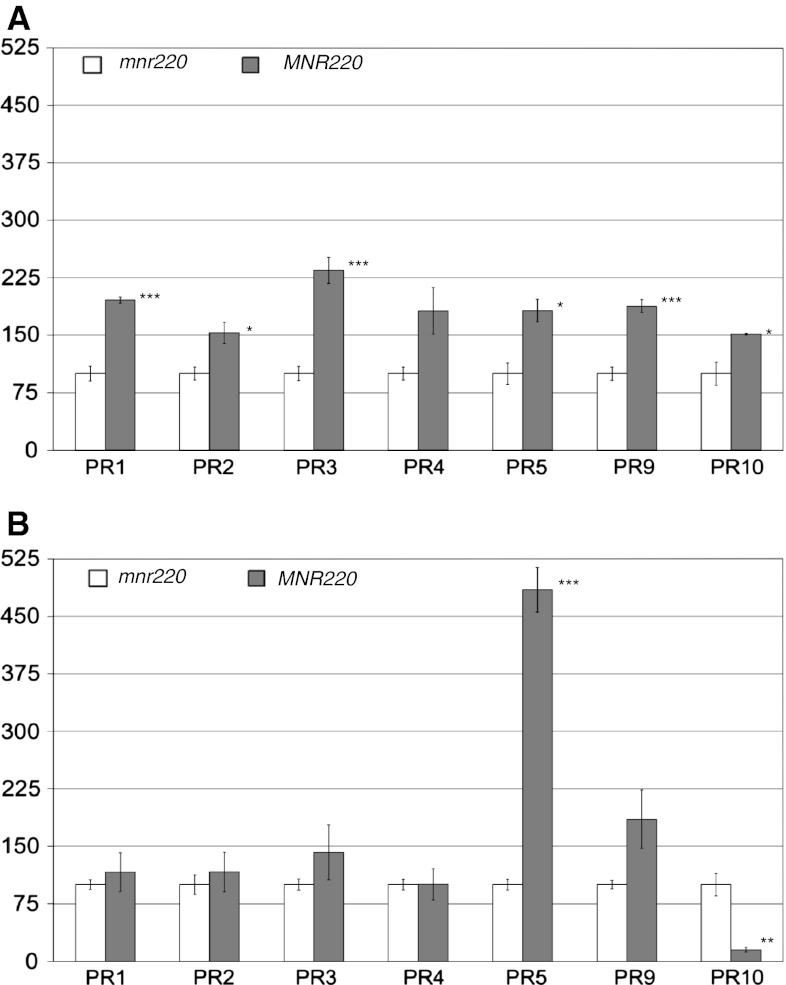
*PR* gene transcript abundance profiles. **a** RT-qPCR *PR* transcript abundance at seedling stage at 0 time point of PBJL inoculation. **b.** RT-qPCR *PR* gene transcript abundance at adult plant stage in the absence of pathogens. Transcript abundance, *y*-axis, given as percent abundance normalized to *mnr220*. *Error bars* represent standard deviation between biological replicates. Mean percent transcript abundance calculated using the ∆∆*C*_t_ method between biological replicates ± standard deviation. Unpaired two-tailed students *t* test between mean of mnr220 and MNR220 and statistical significance indicated as *0.025 ≤ *p* < 0.05, **0.01 < *p* < 0.025, ***p ≤ 0.01

In order to understand how the mutation in MNR220 altered the defense response regulation, we assessed the expression profiles of seven *PR* genes in near isogenic lines (NILs) with either homozygous *MNR220* or *mnr220* alleles derived from one selected heterozygous M_7_ plant after eight generations of selfing and PBJL testing (Fig. 1, for details, see “Materials and methods”). The seven *PR* genes were chosen based on their involvement in defense responses to fungal pathogens in wheat (Desmond et al. 2008) and the primers were designed based on wheat sequence and are able to amplify all three orthologous copies of each *PR* gene in hexaploid wheat genome (data not shown). The transcript abundances were measured using quantitative reverse transcription-polymerase chain reaction (RT-qPCR). To estimate the basal expression levels of the *PR* genes in the NILs, we measured the gene transcript abundances in leaf tissues collected immediately after PBJL inoculation (0 time point) in both two leaf stage seedlings and adult plant flag leaves. The RT-qPCR results revealed similar patterns of transcript abundances for four of the seven selected *PR* genes at the seedling (Fig. 6a) and the adult plant (Fig. 6b) stages. These four *PR* genes (*PR1,* *PR2*, *PR3*, and *PR9*) showed higher levels of transcript abundances in resistant *MNR220* than in susceptible *mnr220* NIL at both the seedling and adult plant stages. The differences between the *MNR220* and *mnr220* were more significant at the seedling stage than those at the adult plant stage for *PR1*, *PR2* and *PR3* and less significant for *PR9* at both stages (Fig. 6a, b). Transcript abundances of *PR5* in adult plants were approximately fivefold higher in the *MNR220* NIL compared to *mnr220*. Similar transcript abundances of *PR4* were detected between the NILs at adult stage, but were approximately twofold higher in the *MNR220* NIL than *mnr220* at the seedling stage. In contrast, transcript abundances of *PR10* were higher in the *MNR220* NIL at the seedling stage, but lower in *mnr220* at the adult plant stage (Fig. 6a, b).

**Fig. 7:**
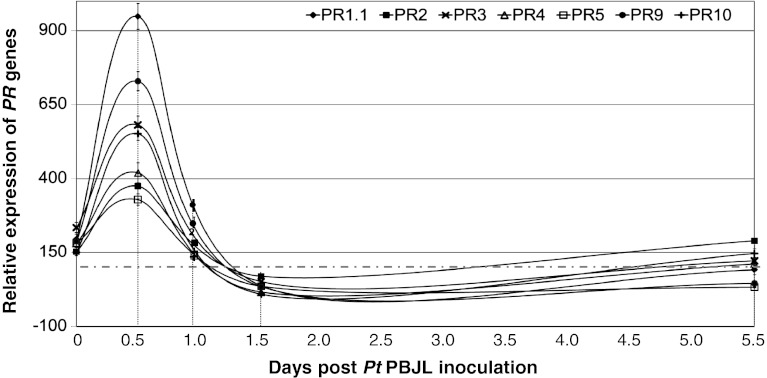
*PR* gene transcript abundance profiles during leaf rust disease development. RT-qPCR *PR* gene transcript abundance in response to PBJL at seedling stage. Transcript abundances in *MNR220* were normalized to *mnr220* at corresponding time point. *Error bars* represent standard deviation between biological replicates

In order to analyze pathogen-induced changes in the seven *PR* gene expression levels during disease development, we measured transcript abundances at five time points post pathogen inoculation. The measurements were taken from leaves collected from bulks of five plants at 0, 0.5, 1, 1.5 and 5.5 dpi and averaged over two independent biological replications. Figure 7 shows the differences between *MNR220* and *mnr220* at the levels of transcript abundances of the seven *PR* genes*.* The basal expressions of all seven *PR* genes in the *MNR220* NIL were a little higher than those in *mnr220* which was normalized as 100 and indicated by a horizontal dash line. The transcript abundances of all seven *PR* genes peaked at 0.5 dpi (Fig. 7), suggesting the largest difference between the NILs was at 0.5 dpi. The differences between the NILs were insignificant at 1.0 dpi (Fig. 7), suggesting the transcript abundances of the *PR* genes in the *mnr220* NIL have increased to similar levels as in the MNR220 NIL, but were 12 h delayed (Fig. 7). At 1.5 dpi, the relative expressions of *PR* genes were all below the dash line, indicating the transcript abundances of the *PR* genes in the *MNR220* NIL were lower than those in the *mnr220* NIL. At 5.5 dpi, the relative expressions of *PR* genes were all close to the dash line, indicating the transcript abundances of the *PR* genes in the *MNR220* NIL were similar to those in the *mnr220* NIL. This preliminary gene expression analysis indicates that these *PR* genes in the *MNR220* NIL were activated and responded to the pathogen earlier after infection than in the *mnr220* NIL.

## Discussion

### A new seedling slow-rusting allele created in MNR220

Rust resistance genes have two modes of action determined by pathogen interaction and are either race specific or race non-specific. Characteristics of race non-specific resistance include the “slow-rusting” resistance features, longer latent period, and decreased uredinial size and number (Dyck 1987; German and Kolmer 1992; Singh and Gupta 1992; Singh et al. 1998). There are known slow-rusting stem (*Sr2*), leaf (*Lr34*, *Lr46*, and *Lr67*), and stripe (*Yr18*) rust resistance genes. *Sr2* is associated with a dark pigmentation trait, called pseudo-black chaff (PBC), expression of which is modified by both biotic and abiotic stresses (Kota et al. 2006). Durable resistance has been achieved in cultivars with *Sr2* and is enhanced by minor genes (Dyck 1987). The resistance conferred by *Lr34* has been well studied (Dyck et al. 1994; Singh and Rajaram 1992; Singh and Gupta 1992). The *Lr34* gene encodes a putative ABC transporter and confers non-hypersensitive resistance to multiple fungal pathogens in wheat (Krattinger et al. 2009). *Lr34* is characterized as an adult plant resistance gene because of the compatible interaction with the pathogen at the seedling stage although seedling resistance of *Lr34* can be induced by low temperature (Pretorius et al. 1993).

Alpowa is a soft, white, semi-dwarf, spring wheat cultivar released by Washington AES/USDA-ARS in 1994. Since its release, Alpowa was reported to carry the rust resistance genes *Lr3a*, *Yr39*, and *YrAlp* but carries neither *Lr34* nor *Sr2*. *Lr3a* does not confer resistance to the primary race *Pt* PBJL utilized in this study. The stripe rust resistance gene *Yr39* confers high-temperature adult plant (HTAP) and *YrAlp* confers all-stage resistance to a few old US *Pst* races (Lin and Chen 2007).

The resistance conferred by *MNR220* has the characteristics of a slow-rusting resistance gene to leaf rust race PBJL at the seedling stage with a 10-day latency period increase, but complete resistance at the adult plant stage to leaf and stripe rusts and powdery mildew. MNR220 showed an early HR-like response to leaf rust and powdery mildew at the seedling and the adult plant stages, but the seedling resistance was compromised after 18 dpi, whereas the adult plant resistance remained effective. These developmental stage-dependent responses were further described in the gene expression profiles of seven *PR* genes at both the seedling and adult plant stages. The profiles revealed elevated expression of four *PR* genes in the absence of infection and a 12 h earlier increase of all seven *PR* genes after pathogen attack in *MNR220* compared to *mnr220*. These results suggest that the mutation changes the regulation of the defense response leading to higher basal defense to certain fungal pathogens, but this basal defense response was eventually overcome by the pathogens at the seedling stage. Two *PR* genes showed different expression profiles between the NILs of *MNR220* and *mnr220* at the adult plant stage. Basal transcript abundance of *PR5* was fivefold higher in MNR220 at the adult plant stage. The *PR5* gene family encodes proteins with homology to thaumatin, osmotin and NP24 which are known to rapidly accumulate to high levels in response to biotic or abiotic stress (Velazhahan et al. 1999). This group of proteins has antifungal activity either by causing fungal membrane lysis at high concentrations or cell membrane leakage at low concentrations (Velazhahan et al. 1999). Transgenic tobacco, potato, and rice over-expressing *PR5* showed higher resistance to several fungal pathogens (Velazhahan et al. 1999). It is unclear whether the elevated level of *PR5* alone was sufficient to make the adult plant resistance complete.

### Disease resistance in MNR220 is associated with changes in negative regulation

Genetic and gene expression analysis revealed that MNR220 activates expression of five *PR* genes that were suppressed in MNR220 in the absence of pathogens. Suppression of *PR* gene expression is a negative regulation, which is thought to be an important mechanism to avoid inappropriate defense responses that are metabolically expensive, and may result in yield penalties to the plants (Brown 2002).

There are three possible mutational events that can change the negative regulation. The first possibility is a loss-of-function event where the mutation disrupts a wild-type negative regulator of resistance. A number of negative regulators of cell death have been identified by the occurrence of spontaneous necrotic lesions in mutants with inactivated regulators (Dangl and Jones 2001). These “lesion mimic” mutants often display increased resistance to pathogens but their mode of action remains unclear as in the *acd1*, *lsd1* and *svn1* mutations described in *Arabidopsis thaliana* (Lorrain et al. 2003; for review see Dangl and Jones 2001; McDowell and Dangl 2003). The mutant MNR220 does not show lesion mimic, but starts leaf senescence earlier than wild-type Alpowa. However, this process is different from the flag leaf tip necrosis associated with the *Lr34* gene (Krattinger et al. 2009), the mutant leaves turn yellow similar to the aging process starting from the oldest leaves, while the flag leaves still maintain green. Alternatively, the mutation could disrupt a pathogen-required host factor. These host factors are negative regulators of disease resistance that are necessary for pathogen invasion. The *Mlo* gene family is an example of a host factor required by *Bgt* for invasion. *Mlo* genes encode heptahelical plasma membrane-localized proteins of unknown function (Büschges et al. 1997; Panstruga 2005).

The second possibility is a gain-of-function event where the mutation creates a new functional resistance allele. This new resistance allele could activate the *R*-gene mediated defense response pathways. Several resistance gain-of-function mutations have been reported including the semidominant mutation *ssi4* (Shirano et al. 2002) and the dominant mutation *snc1* in *Arabidopsis* (Zhang et al. 2003). The gain-of-function mutations in *snc1* and *ssi4* are the result of a constitutive activated R protein induction of defense response.

The third possibility is a loss-of-function event where the mutation disrupts an *R*-gene suppressor. There are several reports of suppressors of rust resistance in wheat (Assefa and Fehrmann 2000; Kerber and Green 1980; Knott 2000; Nelson et al. 1997) where the suppressor suppresses positive regulators of the defense response pathway. These suppressors have been shown to repress the effects of *R* genes located on the A or B genomes of bread wheat (Kerber and Green 1980).

It is unclear, without molecular cloning, whether the *MNR220* allele is a gain-of-function or a loss-of-function allele. With multiple highly conserved orthologous copies of each *PR* gene in hexaploid wheat, it is unknown which orthologs of each *PR* gene transcript abundance was altered by the mutation. However, compared to the *mnr220* NIL, the enhanced transcript levels of the *PR* genes in the mutant suggest the disease resistance in MNR220 is associated with changes in negative regulation in the defense pathways.

### Resistance to multiple fungal pathogens is likely associated with a single locus in MNR220

The mutation rate in the EMS-mutagenized population used in this study was estimated to be 1 in 11.5 kb of DNA (Feiz et al. 2009) which suggests that many mutations occurred in each individual. Several traits mostly from different mutations including plant height, spike morphology, and tip sterility, segregated in the selfed progeny of MNR220 but none co-segregated with disease resistance. Resistance to leaf rust, stem rust and powdery mildew co-segregated in total 106 F_2:3_ lines derived from two crosses. It is therefore probable that resistance to multiple fungal pathogens in MNR220 is conferred by a single locus. Single gene multi-pathogen resistance is consistent with evidence suggesting limited sets of defense-related molecules in each host are used to defend against highly diverse pathogens (Dangl and Jones 2001), although more genes are likely to be involved in either enhancing or reducing the levels of defense response evidenced by our observation that F_1_ individuals from the cross of Chinese Spring/MNR220 were more resistant to PBJL than those of cross of MNR220/Alpowa. This also supports the findings that even evolutionarily diverse pathogens target similar host defense immunity machinery (Mukhtar et al. 2011). In addition to our report here, one other mutant plant conferring resistance to multiple rust pathogens has been reported in wheat (Boyd et al. 2006). Only large scale progeny testing and molecular cloning of the gene(s) will reveal the true basis of the MNR220 mutation. Molecular analysis of the disease resistance locus in MNR220 may lead to a better understanding of the regulation of defense response networks in wheat.
